# The Impact of Intrauterine Devices on the Risk of Ureaplasma Species Overgrowth: A Review of the Literature

**DOI:** 10.7759/cureus.92829

**Published:** 2025-09-21

**Authors:** Julia L Armstrong, Madison Uzwy, Jessica N Smock, Joyce R Miller

**Affiliations:** 1 Osteopathic Medicine, Nova Southeastern University Dr. Kiran C. Patel College of Osteopathic Medicine, Fort Lauderdale, USA; 2 Obstetrics and Gynecology, Baptist Health South Florida, Miami, USA

**Keywords:** iud, mycoplasma, obgyn, opportunistic, overgrowth, species, ureaplasma, ureaplasma parvum, ureaplasma urealyticum, urogenital

## Abstract

*Ureaplasma* species (*Ureaplasma* spp.) are commensal organisms of the lower urogenital tract, but overgrowth has been associated with vaginal infections such as bacterial vaginosis (BV) and sequelae, including pelvic inflammatory disease (PID), infertility, and pregnancy complications such as preterm labor. Intrauterine devices (IUDs) are an effective contraceptive method; however, their use has been linked to an increased risk of urogenital infections, including those involving *Ureaplasma* spp., which may disseminate and lead to adverse health outcomes. There is still much uncertainty surrounding the connection between *Ureaplasma* spp. and IUDs, calling for further research to analyze the possible associated adverse effects. This literature review aims to provide a comprehensive overview of the relationship between IUDs and the risk of *Ureaplasma* spp. overgrowth. It addresses factors such as pathogenicity, the connection between IUDs and infection risk, screening before IUD insertion, diagnostic challenges of *Ureaplasma* spp., treatment options, and implications in fertility and pregnancy.

## Introduction and background

Ureaplasma

*Ureaplasma* species (*Ureaplasma* spp.) were first discovered in 1954 with the use of agar cultures containing samples of urethral exudates from male patients with nongonococcal urethritis [1]. *Ureaplasma* spp. have been detected as the most common *Mycoplasma* found in the urogenital tracts of both males and females [2].

Although *Ureaplasma *spp*.* are considered normal inhabitants of the urogenital flora, they have been implicated in a variety of opportunistic infections. When overgrowth occurs, *Ureaplasma *spp. have been associated with a decrease in *Lactobacillus* abundance in the vaginal flora, as well as an increased incidence of bacterial vaginosis (BV), heightened risks in pregnancy, and infertility [3,4]. Due to the high rate of *Ureaplasma* *parvum *(*U. parvum)* colonization in asymptomatic women (30%), *Ureaplasma* spp. are not commonly tested for or treated unless there is a prior or current history of BV [4]. In a 2015 study on the comparison of the frequency of the two serovars, significantly more women 25 years and younger were found to be infected with *Ureaplasma urealyticum* (*U. urealyticum) *(23.4%) compared to those over the age of 25 (9.2%) [3]. Although *Ureaplasma* spp. and *Mycoplasma* share many characteristics, including their associations with urogenital infections and lack of a cell wall, *Ureaplasma* spp. were placed into their own genus within the *Mycoplasmataceae*family in 1974 due to their distinct expression of urease [5]. Additionally, *Ureaplasma* spp. strains have been historically difficult to culture due to their extremely small diameter, ranging from 15 to 25 µm [6]. Through the use of urease, some laboratories judge infection by measuring the change in pH in liquid medium when hydrolysis of urea into ammonia (NH_3_) and carbon gas is produced [6]. While *U. urealyticum *has been commonly associated with urogenital tract infections, the difficulty in differentiating between the two species calls for further evaluation of the impact of *U. parvum* [1].

Intrauterine devices

Intrauterine devices (IUDs) are one of the most commonly used methods of contraception. Studies have shown that regarding pregnancy prevention, the effectiveness of IUDs is closely comparable to that of sterilization or tubal ligation [7].

The advantages of IUDs over sterilization include reversibility and relatively low cost. There are currently two main types of IUDs used in the United States: copper-containing IUDs (failure rate of 0.08%) and levonorgestrel-containing IUDs (failure rate of 0.02%) [8]. While IUDs are an effective method for many women desiring protection against pregnancy, they may be contraindicated in certain instances. Some contraindications may include pregnancy or suspected pregnancy, sexually transmitted infections (STIs), pelvic inflammatory disease (PID), confirmed or suspected malignancy originating in the uterus or cervix, and abnormal uterine bleeding [8].

One of the most serious but rare complications of IUD insertion is uterine perforation, occurring in less than one in 1000 insertions [9]. Additional complications may include ectopic pregnancy and an increased risk of infections such as PID. A study of 236 reproductive-age women in Croatia assessed the risk of opportunistic bacteria, including *Escherichia coli (E. coli)* and *U. urealyticum*, in IUD vs. non-IUD users. Infection and colonization rates of both bacteria were found more frequently in IUD users (p < 0.001) when compared to the control group [9]. Due to the increased risk of opportunistic infections in IUD users, additional research is needed to combat the possible negative impact of dissemination and complications among this patient population. Increased global awareness of *Ureaplasma* spp. is critical in clinical practice due to the possible negative implications for pregnancy outcomes and vaginal health.

## Review

Search strategy & databases

In our literature search on the risk of IUD use and *Ureaplasma* spp. overgrowth, we utilized multiple databases, including PubMed, Embase, Web of Science, JSTOR, and Ovid MEDLINE. The search was limited to articles published between 2000 and 2025. Keywords included "*Ureaplasma*", "intrauterine device", "*Mycoplasma*", "overgrowth", "species", "urogenital", and "opportunistic". To further organize, we used the EndNote reference manager to properly cite references. This is a narrative review. Due to the variability in the included articles, no ​​quantitative synthesis was performed. Rather, data were synthesized in a narrative design to highlight major findings.

Eligibility criteria

Studies were eligible for inclusion if they analyzed *Ureaplasma* spp. in the context of IUD use, published between 2000 and 2025, and were available in English. We included randomized control trials (RCTs), observational studies, and case series involving women of reproductive age and pregnancy outcomes. Exclusion criteria included articles published prior to 2000, non-peer-reviewed articles, and case reports due to their narrow scope.

Screening and study selection

We screened studies by reviewing abstracts and further eliminating irrelevant ones before completing full-text analyses of the included literature. Each author contributed to the literature selection, grammar corrections, and revisions. Bi-weekly Zoom (Zoom Video Communications, San Jose, CA) meetings were conducted to discuss literature review objectives, additions, and deletions. After multiple edits, a final draft was created with the input and evaluation of each author prior to submission. This study’s primary objective focused on the impact of IUDs on the risk of *Ureaplasma* spp. overgrowth.

Ureaplasma: pathogenicity and clinical relevance

The *Mycoplasmataceae* family is composed of bacteria lacking a cell wall and is broken up into two different genera, *Mycoplasma* and *Ureaplasma* [6]. The main distinguishing feature that separates *Ureaplasma* from *Mycoplasma* is their ability to produce urease, an enzyme that converts urea into ammonia and carbon dioxide [10]. The conversion of urea into ammonia and carbon dioxide increases the vaginal pH, creating a more alkaline environment. As a result, infections with *Ureaplasma *spp.are often associated with a higher genital tract pH, which is one of the predictors of BV according to Amsel’s criteria [11]. The elevated pH seen in BV is typically attributed to excessive growth of *Gardnerella vaginalis* and reduced growth of *Lactobacillus *species* (Lactobacillus *spp.); however, recent studies have demonstrated that genital tract colonization with *U. urealyticum *and *U. parvum* quadruples the risk of acquiring BV [11,12].

Beyond urease activity, *Ureaplasma *spp. possess several other virulence factors, including immunoglobulin A (IgA) protease, phospholipases A and C, hemolytic activity, and host cell attachment [6].

The *Ureaplasma* genus has been divided into two separate species, *U. urealyticum* and *U. parvum,* based on differences in serovars, which are subtypes of a bacterial species distinguished by the bacteria’s surface antigens [13]. *U. urealyticum* includes serovars 2, 4, 5, 7, 8T, 9, 10, 11, 12, and 13, while *U. parvum* consists of serovars 1, 3, 6, and 14 [13,14]. Since *U. urealyticum* has a larger genome than *U. parvum*, it has been speculated that *U. urealyticum* may have a higher pathogenicity than* U. parvum* [15]. This idea is still under debate, as a study published in 2011 demonstrated that there is extensive horizontal gene transfer between the two *Ureaplasma* spp., indicating that genes do not stay within their original serovar groups [16]. This would indicate that a strain originally designated as one serovar may acquire genes from another serovar and could therefore alter its antigenic profile [15]. This alteration may lead to misidentification and unreliable serotyping between species in a clinical setting.

*Ureaplasma *spp. have been found to colonize anywhere from 40% to 80% of sexually mature asymptomatic women [17]. Despite this, *Ureaplasma *spp. have also been implicated in a range of pathological conditions, including non-gonococcal urethritis, prostatitis, urinary stones, PID, infertility, preterm birth, premature rupture of membranes, neonatal pneumonia, and neonatal respiratory distress syndrome [18,19]. Because these organisms have been isolated from the lower genital tract in both healthy and symptomatic individuals, they are considered opportunistic pathogens [2].

Younger women, particularly those under the age of 25 and those with an earlier age at first intercourse, exhibit a higher prevalence of *U. urealyticum* infections [20]. Additionally, the risk of *U. urealyticum* infection correlates with an increased number of sexual partners, as well as factors such as smoking and a history of induced abortion [2]. HIV-positive individuals also demonstrate a higher incidence of *Ureaplasma* spp. infections, as do patients with a history of infectious kidney stones [21].

PID is defined as inflammation and infection of the upper genital tract affecting the uterus, fallopian tubes, and/or ovaries [22]. PID often leads to tubal factor infertility, which accounts for a large proportion of female infertility cases [23]. Historically, PID has been largely attributed to complications from long-standing *Chlamydia trachomatis* (*C. trachomatis*) and *Neisseria gonorrhoeae* (*N. gonorrhoeae*) infections; however, the etiology of up to 70% of cases is unknown [24]. *Ureaplasma* spp. have been isolated from affected fallopian tubes in patients with PID and found modest associations with PID sequelae such as endometritis [24]. Although there is no conclusive association between PID-induced infertility and *Ureaplasma *spp., one study demonstrated that *Ureaplasma* spp. was detected in 20.8% of infertile female participants [25]. The exact mechanism behind this association remains unclear, but it is hypothesized that infection-related inflammation may disrupt normal immune system modulation, impairing fertilization and embryo implantation [26].

Ascending bacterial infections contribute to around 40% of spontaneous preterm births, with *Ureaplasma *spp. being the most commonly isolated organism in the amniotic fluid of preterm pregnancies [27,28]. A study found that preterm labor occurred in 58.6% of *U. urealyticum*-positive patients, while only 4.4% of patients negative for *U. urealyticum* experienced preterm labor [29].

An additional study analyzed various women who underwent cesarean delivery between 23 and 34 weeks of gestation. They found that 43.9% of individuals who underwent cesarean due to preterm labor or premature rupture of membranes were colonized with *U. urealyticum *[19]. In contrast, only 2.7% of individuals who had a cesarean for other indications tested positive for *U. urealyticum *[19]. This study highlights the association between intrauterine colonization with *U. urealyticum* and preterm labor as well as premature rupture of membranes [19]. These studies highlight the clinical importance of the use of polymerase chain reaction (PCR) testing of amniotic fluid during the second trimester for *U. urealyticum,* which may help identify women at risk for subsequent preterm labor and birth [29].

Intrauterine devices and risk of infection

The use of IUDs as a means of contraception has been gradually increasing with a 6.2% annual increase between the years 2006 and 2017 [30]. Although IUD use is associated with multiple health benefits, including reduced risk of ovarian cancer, treatment of complex atypical hyperplasia and low-risk endometrial cancer, and high contraceptive efficacy, these devices are also linked to potential complications [30,31].

The most common complication is displacement or accidental removal of the IUD after insertion [8]. There is also a very mild risk of unwanted pregnancies with IUD use, which falls around 0.08% for the copper IUD and 0.02% for the 20 mg levonorgestrel IUD [32]. One of the most severe complications is the risk of uterine perforation during insertion; however, this risk is low [33]. Another serious complication of hormonal IUDs is a mild association with an increased risk of developing breast cancer [34].

One of the most concerning complications for healthcare providers is the possibility of developing PID; however, this risk is low and estimated to be seen in less than 1% of IUD users [35]. Although women with subclinical *N. gonorrhoeae *or *C. trachomatis* who undergo IUD insertion have an increased risk of developing salpingitis compared to those without these infections, studies suggest that this risk is comparable to that of infected individuals who do not have an IUD [36].

**Figure 1:**
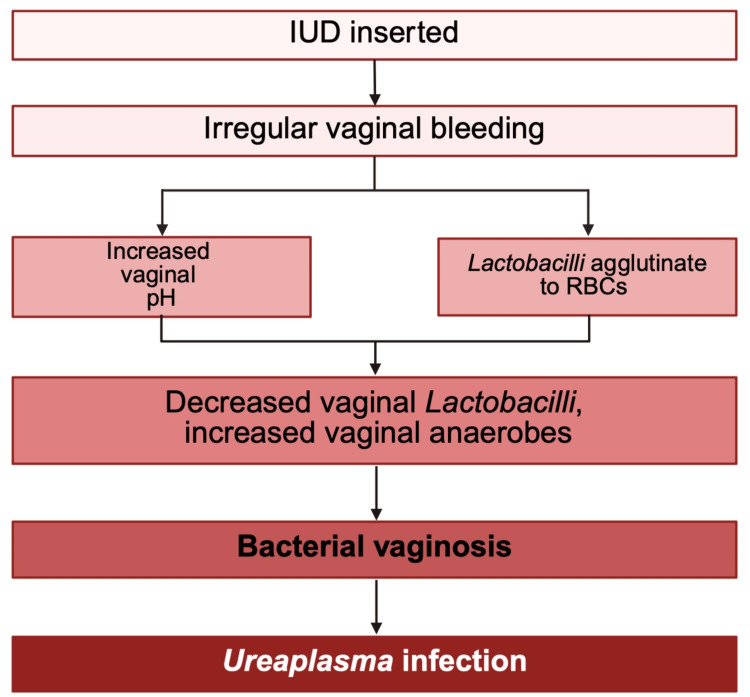
Proposed mechanism of Ureaplasma infection in IUD users. IUD: intrauterine device; RBC: red blood cells. Figure created with BioRender.com.

Studies have demonstrated an increased risk of BV and associated microbiota, including *Gardnerella vaginalis* and *Atopobium vaginae*, linked to the use of the copper IUDs, as compared to other hormonal contraceptive methods, not including the hormonal IUDs [37]. As shown in Figure 1, one proposed theory to explain this connection is the association between copper IUDs and irregular bleeding, which typically presents as an increased volume and duration of menstrual flow and could facilitate the growth of anaerobic bacteria in the vagina [38]. Another study examined the relationship between BV and hormonal IUDs, finding that the increased risk was present among users with irregular bleeding. This suggests that irregular bleeding may represent the causal pathway linking BV and IUD use [38].

There are multiple theories as to why bleeding may increase rates of BV. One idea is that menstrual blood raises the pH of the normally acidic environment present in the vagina [38]. This increase in pH can promote the growth of anaerobic bacteria typically associated with BV and impedes the growth of beneficial aerobes normally present in the vaginal microflora, such as *Lactobacillus *spp. Additionally, *Lactobacilli* tend to agglutinate onto red blood cells, so when more blood is present, the levels of vaginal *Lactobacilli* decrease [38].

Another factor that has been associated with an increased risk of infection in IUD users is that IUDs, similar to other medical implants, can serve as a site for biofilm formation [39].

Because of the alterations in the vaginal microflora of IUD users, certain opportunistic infections have been associated with this form of contraception. A study conducted in 2013 showed that individuals with IUDs had a higher prevalence of *U. urealyticum* [20]. A third study found that patients experiencing vaginal discharge with an IUD had a significantly higher rate of *U. urealyticum* infection than those experiencing vaginal discharge without an IUD [40]. This emphasizes the importance of screening for asymptomatic vaginal and cervical infections before IUD insertion.

IUDs and local immune modulation

As with the insertion of any foreign body, IUD insertion has the potential to negatively impact the human microflora and immune environment. A 2016 study found an increase in inflammatory cytokines, including tumor necrosis factor-α (TNF-α), interleukin-1β (IL-1β), and interferon-γ (IFN-γ) in the endocervix and endometrium of women with hormonal IUDs when compared to the control group of non-IUD users [41].

Progesterone release is another important factor affecting the immune environment of patients with IUDs. Progesterone acts as an immunosuppressant by inhibiting mast cell degranulation and increasing levels of T helper type 2 (Th2) cells [42]. Th2 cells increase levels of mucus production [42]. Considering the heightened growth of *Ureaplasma *spp. in cervical mucus (49%) compared to vaginal fluid (34%), increased cervical mucus production may act as an opportunistic environment for *Ureaplasma *spp*.* [43]. The various microbiome changes in patients using IUDs may be a contributing factor to facilitating *Ureaplasma* spp. colonization and overgrowth.

One study followed 17 women four weeks post insertion of either a levonorgestrel-containing IUD or a copper-containing IUD. After analyzing vaginal samples collected via swabbing, significant increases were noted in levels of IL-1β (p = 0.018), interleukin-6 (IL-6) (p = 0.046), and TNF-α (p = 0.029) among IUD users [44]. Furthermore, a 2009 experiment discussed the role of *U. parvum* in rodent models in which rodent groups were inoculated with different amounts of rodent-adapted strains of *Ureaplasma* spp., including 10^9^, 10^3^, 10^5^, 10^7^, or 10^10^ [45]. It was found that rodents inoculated with ≥ 10^7^ of *U. parvum* remained infected after two weeks with* U. parvum* (p < 0.04) and had a higher incidence of urinary tract infections (UTIs), suggesting overgrowth as an infection source [45]. Researchers concluded that complications from *Ureaplasma* spp. overgrowth are strongly correlated to host-specific immune factors, including an increase in pro-inflammatory cytokines such as IL-6 and IL-8 [45]. Considering the prior discussion of IUDs causing an increase in pro-inflammatory cytokines, more research is necessary to determine the connection between IUDs and *Ureaplasma* spp. overgrowth [45].

Inflammatory response to Ureaplasma

**Figure 2:**
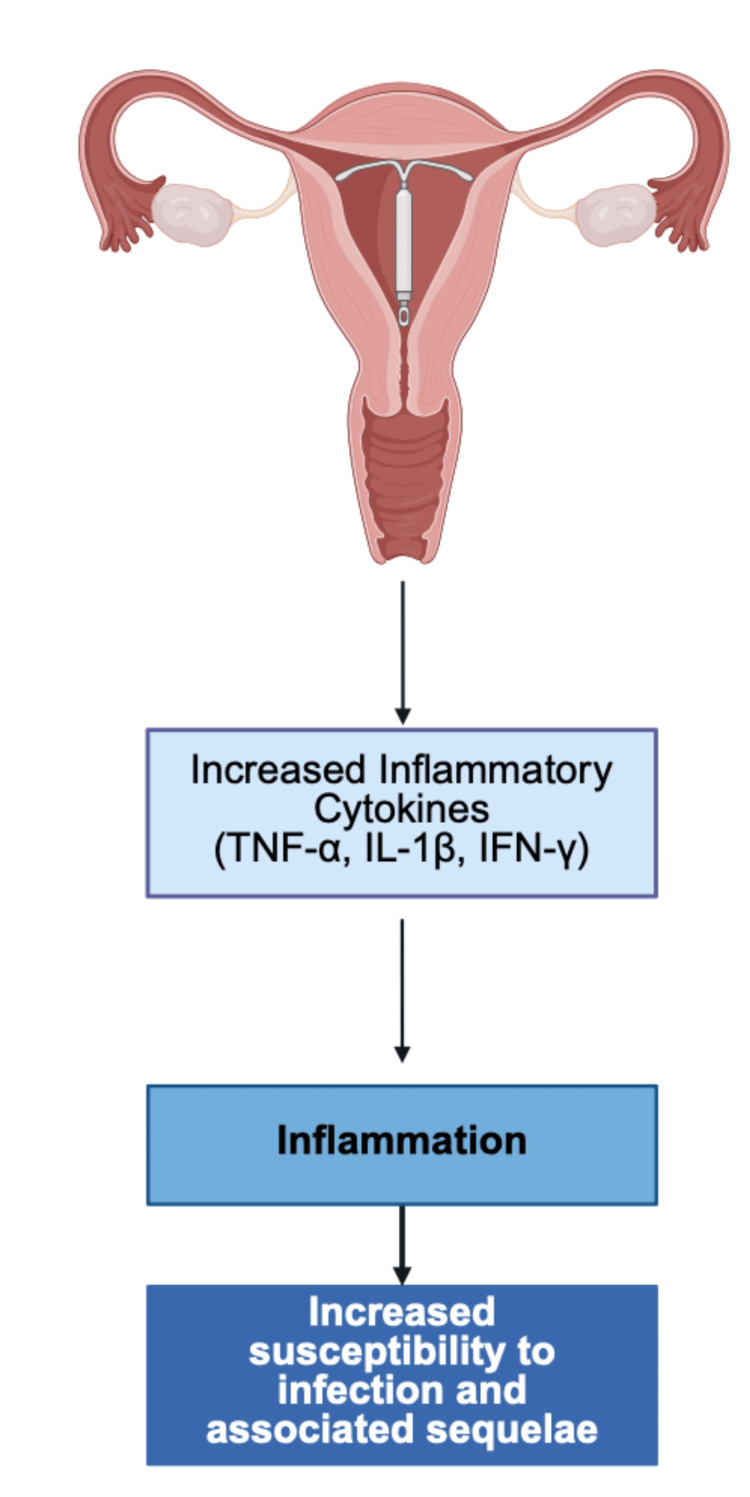
Proposed mechanism of inflammatory response and IUDs. IUDs: intrauterine devices; TNF-α: tumor necrosis factor-α; IL-1β: interleukin-1β; IFN-γ: interferon-γ. Figure created with BioRender.com.

While the mechanism of *Ureaplasma* spp. growth is still not fully understood, several virulence factors, including IgA protease, phospholipase A, and phospholipase C, have been identified [6]. Considering the high level of mucus in the cervix, it may be a common reservoir for overgrowth. In response to *Ureaplasma* spp. antigens, the local immune response induces inflammation with high levels of cytokines, including IL-6 and IL-8 [6]. The chronic low-grade inflammation associated with *Ureaplasma *spp. may be implicated in various diseases relating to uterine health. For example, early development of endometriosis is associated with tissue breakdown due to local inflammatory mediators such as IL-6 and TNF-α [46]. The proliferation of endometrial tissue and subsequent fibrosis is mediated by Th2 and T regulatory cells, which are both upregulated in the presence of progesterone [46]. This may suggest the further impact that IUD insertion has on both *Ureaplasma* spp. and its various complications. This proposed mechanism may be seen in Figure 2.

Screening before IUD insertion

While the American College of Obstetricians and Gynecologists (ACOG) recommends screening female patients who fall under the category of “high-risk for sexually transmitted infections,” there is currently no protocol in place for STI screening before IUD insertion [47]. Due to the association between *Ureaplasma *spp., *C. trachomatis*, and *N. gonorrhoeae* with PID, pre-insertion screening may decrease the dissemination of infection and long-term sequelae. Furthermore, considering PID is a contraindication to IUD insertion, pre-screening for the causative organisms should be required [47]. Since *C. trachomatis* and *N. gonorrhoeae* are often asymptomatic, an unknown infection at the time of insertion may act as a gateway to PID, which has been noted to occur in 30% of the time in patients diagnosed with these two organisms [48]. If left untreated, PID may cause future ectopic pregnancy and infertility. Testing of these organisms is relatively quick. Requiring under 24 hours to diagnose, *C. trachomatis* and *N. gonorrhoeae* are detected via urine, vaginal, or cervical specimens with nucleic acid amplification testing (NAAT) [49].

Challenges in diagnosing

There is currently no evidence to support testing for or treating *Ureaplasma* spp. in asymptomatic individuals; therefore, routine testing and treatment are not currently recommended [50]. Many individuals serve as asymptomatic carriers, and the vast majority do not go on to develop clinical disease [50].

The gold standard for diagnosing *U. urealyticum* is bacterial culture using 10B medium, which detects urease activity [51]. The bacteria release ammonium ions, resulting in the alkalinization of the medium and a corresponding color change [51]. On A8 agar, *U. urealyticum* and other *Mycoplasma* species exhibit a characteristic "fried egg" colony morphology [52]. However, *Ureaplasma *spp. colonies are distinguishable by their unique golden, "brown sea urchin-shaped" appearance [52].

While culture techniques can reliably identify *Ureaplasma* spp., PCR is necessary to distinguish between the species *U. urealyticum* and *U. parvum* [53]. An added advantage of PCR is its ability to quantify the amount of *Ureaplasma* spp. DNA, which correlates with bacterial load [53]. This quantitative data may serve to differentiate between asymptomatic low-load carriers and symptomatic individuals with higher bacterial loads, suggesting a potential relationship between load intensity and clinical manifestation [54].

Up to 60% of individuals with a *Ureaplasma* spp. infection have sexual partners who also test positive for the organism, indicating a potential for re-infection [55]. In such cases, concomitant treatment of both the infected individual and their partner(s) may be necessary to effectively eradicate the infection [55]. This presents a challenge to some, as fear of embarrassment or scrutiny may provide a significant barrier to proper treatment.

Treatment options and resistance patterns

First-line treatment for *Ureaplasma* spp. includes oral azithromycin 1000 mg as a single dose or oral doxycycline 100 mg twice daily for one week, which are of comparable therapeutic effect [2,56]. Oral erythromycin 500 mg for one week is offered to those who do not respond to doxycycline due to resistance [39]. Fluoroquinolones were also found to have equal efficacy to that of doxycycline [39]. Studies revealed that erythromycin use is associated with the highest resistance rate (2.3%), while doxycycline use is associated with the highest sensitivity (98%) [57,58]. A summary of the treatment options and dosages may be seen in Table 1.

**Table 1:** Summary of treatment regimen for Ureaplasma species. Resistance rates adapted from [57]. Sensitivity rates adapted from [58]. PO: oral administration.

Drug name	Route	Recommended dosage	Recommended use	Resistance (%)	Sensitivity (%)
Azithromycin	PO	1000 milligrams (1000 mg) single dose	First-line therapy	1.3	69.2
Doxycycline	PO	100 milligrams (100 mg) twice daily for one week	First-line therapy	0.8	98
Erythromycin	PO	500 milligrams (500 mg) four times daily for one week	Second-line therapy	2.3	78.1

Impact of IUD removal

The potential impact of IUD removal on the management of *Ureaplasma *spp. infections remains uncertain, despite some cases in which *Ureaplasma* spp. cultures were obtained from IUDs in affected individuals [59]. Although it has been thought that removing the IUD at the initiation of treatment may eliminate a possible source of infection and facilitate a faster recovery, there is limited evidence to substantiate this theory [20]. However, women with IUDs tend to experience higher rates of recurrence, regardless of their partner’s treatment status [56].

Implications for fertility and pregnancy

As fertility challenges continue to affect an increasing number of women, the demand for effective diagnostic and therapeutic solutions has risen [60]. Studies have shown that *Ureaplasma* spp. are present in 32% of fertile women, whereas infertile women exhibit a significantly higher culture positivity rate of 55% [2]. Specifically, *U.* *urealyticum* has been identified in 32% of women with infertility [61]. Moreover, *Ureaplasma* spp. infections are notably more prevalent among women who experience spontaneous abortions, stillbirths, and premature births, compared to those with healthy, full-term pregnancies [62]. Preterm birth rates are significantly higher in *Ureaplasma *spp*.*-positive women when compared to their *Ureaplasma *spp*.*-negative counterparts [29].

Among very low birth weight infants, those born with *Ureaplasma *spp. infection are twice as likely to develop chronic lung disease or succumb to mortality compared to uninfected infants of similar weight [63]. Furthermore, colonization by *U. urealyticum* in neonates has been associated with an increased risk of retinopathy of prematurity, further highlighting the potential complications linked to this infection [64].

## Conclusions

Although *Ureaplasma* spp. is a bacterium commonly found in the vaginal flora, overgrowth has been associated with bacterial vaginosis, pelvic inflammatory disease, infertility, and adverse pregnancy outcomes. IUDs may predispose individuals to *Ureaplasma* spp. infections as they alter the vaginal microbiome. This literature review highlights the potential association between IUD use and increased risk of *Ureaplasma *spp*. *infection in reproductive-age women, emphasizing the necessity for further awareness and additional screening measures. As STI screening is already offered to high-risk populations, it raises the question as to whether additional *Ureaplasma* spp. testing would be beneficial in these individuals, especially those who undergo a partner change. Additional questions may include whether it would be beneficial to retest patients who possess IUDs after treatment due to the possibility of recurrence, and after how many recurrences should IUD removal be considered. The lack of standardization for diagnosis in symptomatic patients again reiterates the need for additional research in this area.
